# Targeted resequencing analysis of 31 genes commonly mutated in myeloid disorders in serial samples from myelodysplastic syndrome patients showing disease progression

**DOI:** 10.1038/leu.2015.129

**Published:** 2015-06-26

**Authors:** A Pellagatti, S Roy, C Di Genua, A Burns, K McGraw, S Valletta, M J Larrayoz, M Fernandez-Mercado, J Mason, S Killick, C Mecucci, M J Calasanz, A List, A Schuh, J Boultwood

**Affiliations:** 1 LLR Molecular Haematology Unit, Nuffield Division of Clinical Laboratory Sciences, Radcliffe Department of Medicine, University of Oxford, Oxford, UK; 2 NIHR Biomedical Research Centre, University of Oxford, Oxford, UK; 3 H. Lee Moffitt Cancer Center and Research Institute, Tampa, FL, USA; 4 Department of Genetics, University of Navarra, Pamplona, Spain; 5 Department of Haematology, Royal Bournemouth Hospital, Bournemouth, UK; 6 Hematology and Bone Marrow Transplantation Unit, University of Perugia, Perugia, Italy

The myelodysplastic syndromes (MDS) are clonal disorders of the hematopoietic stem cell, characterized by ineffective hematopoiesis and peripheral blood cytopenias, and patients typically have a hypercellular bone marrow. Approximately 30–40% of patients undergo leukemic transformation to acute myeloid leukemia (AML) during the course of their disease.^1^ Several recurrent gene mutations have been identified in MDS using next-generation sequencing (NGS), and recent studies have greatly illuminated the molecular landscape of this disorder.^2, 3, 4^ However, the molecular events driving MDS progression to AML remain poorly understood.

To investigate the genetic basis of disease progression in MDS, and in particular of leukemic transformation to AML, we evaluated the frequency and chronology of the acquisition of a large number of gene mutations using a targeted NGS myeloid gene panel on serial (paired) bone marrow samples from 41 MDS patients before (preprogression) and after progression (postprogression) to a more advanced subtype (*n*=5) or to AML (*n*=36) (Supplementary Table 1). The mutational profile was characterized using a TruSeq Custom Amplicon (TSCA) panel (Illumina, San Diego, CA, USA), a development of our previously reported myeloid gene panel,^5^ targeting the hotspots of 31 recurrently (>1%) mutated genes in myeloid malignancies (Supplementary Table 2). Dual-barcoded TSCA libraries were sequenced on an Illumina MiSeq platform, and variants were annotated and filtered using Illumina VariantStudio (Supplementary Methods). The proportion of sequencing reads supporting a given mutation (variant allele frequency, VAF) was used to estimate the fraction of tumor cells carrying that mutation, and to determine whether mutations are clonal (in all tumor cells) or subclonal (in a fraction of tumor cells).

A total of 99 and 122 mutations across 23 genes were identified in preprogression and postprogression samples, respectively (Supplementary Tables 1 and 3). The number of mutations was generally higher in the postprogression samples: the number of cases with one or two mutations was 24 in preprogression samples and 17 in postprogression samples, whereas the number of cases with three or four mutations was 12 in preprogression samples and 17 in postprogression samples (Supplementary Figure 1). These data are consistent with a previous study showing that, in a large MDS patient cohort, leukemia-free survival deteriorated steadily as the number of driver mutations increased, suggesting that transformation to AML is driven by clonal evolution associated with the acquisition of new driver mutations.^3^

The most frequently mutated genes (in >15% of samples) were *ASXL1*, *TET2*, *SRSF2*, *U2AF1*, *RUNX1* and *TP53*. *ASXL1*, encoding an epigenetic regulator, was the top ranking mutated gene with a frequency of 44% in preprogression samples and 46% in postprogression samples (Table 1). In contrast, the splicing factor *SF3B1*, widely reported as the most frequently mutated gene in MDS, was mutated in only two cases in our cohort (5%). Given that the frequency of *ASXL1* mutations in MDS ranges from 11 to 15%, and of *SF3B1* mutations from 20 to 28% in unselected studies,^6^ the mutation data concerning these two genes are clearly strikingly different in our study. It should be noted, however, that the patient cohort used in this present study was highly selected—that is, comprising only patients whose disease had progressed. Our data thus indicate that *ASXL1* mutations are strongly associated with MDS cases that show disease progression to AML and conversely that *SF3B1* mutations are rarely associated with MDS cases that show disease progression. This finding is consistent both with the status of *ASXL1* as a poor prognostic marker in MDS^7^ and with the strong association of *SF3B1* mutation with a good prognosis in MDS and with the low-risk MDS subtype refractory anemia with ring sideroblasts.^8^

In agreement with previous studies,^2, 3, 4, 9^ mutations in splicing factor genes (*SF3B1*, *SRSF2*, *U2AF1* and *ZRSR2*) were mutually exclusive, with the only exception being one case with mutations in both *U2AF1* and *ZRSR2*. Mutations of genes involved in splicing (*SRSF2*, *U2AF1* and *ZRSR2*), chromatin modification (*EZH2* and *ASXL1*) and DNA methylation (*TET2*, *IDH1*/*2* and *DNMT3A*) were present in the preprogression and postprogression samples for almost all cases harboring mutations in these genes, and thus represent early events in the disease course in these cases. Interestingly, age-related clonal hematopoiesis, with the majority of variants occurring in *DNMT3A*, *TET2* and *ASXL1*, has been shown to be a common condition that is associated with increases in the risk of hematologic cancer.^10, 11^ Mutations of genes involved in transcriptional regulation (*RUNX1*, *ETV6* and *PHF6*) and signal transduction (*NRAS* and *KRAS*) were found in the postprogression sample only in the majority of cases, suggesting that these are often late events that may co-operate with early events to drive disease progression (Figure 1). Papaemmanuil *et al.*^3^ similarly showed that mutations in genes involved in RNA splicing and DNA methylation occur early during the disease course in MDS, whereas driver mutations in genes involved in signaling often occur later. However, this study concerned the analysis of different MDS subtypes, not serial samples from the same patients.

Emerging data suggest that key differences in disease phenotype can be driven by different combinations of comutated genes in MDS.^12^ Reasoning that such gene mutation associations may also have a role in disease progression in MDS, we investigated pairwise associations between mutated genes in our study of MDS serial samples. We found that all seven preprogression samples carrying *RUNX1* mutations were also mutated for *ASXL1*, whereas 11 of 34 preprogression samples without *RUNX1* mutations had *ASXL1* mutations (two-sided *P*=0.001, Fisher's exact test). The *ASXL1*–*RUNX1* mutated gene association has been previously shown to be significant in studies on large MDS cohorts.^2, 3^ We also found that all five preprogression samples in our cohort with *ZRSR2* mutations also carried *ASXL1* mutations, whereas 13 of 36 preprogression samples without *ZRSR2* mutations carried *ASXL1* mutations (two-sided *P*=0.011, Fisher's exact test). Co-occurrence of mutations in splicing factor genes and in genes involved in epigenetic regulation has been reported previously;^2, 3^ however, the *ASXL1*–*ZRSR2* association has not been described. In the postprogression samples, there were five cases with mutations of both *NRAS* and *RUNX1* (compared with two cases in preprogression samples), an association reported as significant in a previous MDS study.^3^ We have also observed co-occurrence of *NRAS* and *ASXL1* mutations in five postprogression samples (compared with two cases in preprogression samples) in our study. Interestingly, *NRas* mutation and *Asxl1* loss co-operate to drive myeloid proliferation and myeloid leukemia in mice,^13^ and our data on MDS serial samples support this observation. These co-occurring gene mutations may thus have a role in disease progression in MDS.

Specific associations between mutated genes and chromosomal abnormalities have also been described in MDS.^6^ In our study, we found that *TP53* mutations were present in all four cases in our cohort with abnormalities of chromosome 5, *U2AF1* mutations were present in three of five cases with −20/del(20q) and two of four cases carrying *SETBP1* mutations had −7; these association have been previously reported.^6^

The average VAF of some mutations changed markedly during disease progression, with *TP53* showing the largest average VAF fold increase (>50%) in postprogression samples compared with preprogression samples among genes mutated in more than five cases, suggesting that this mutation had a major role in driving disease progression in these patients (Supplementary Table 4). *RUNX1* showed an average VAF fold increase of approximately 25% in postprogression samples compared with preprogression samples, and interestingly both *RUNX1* and *TP53* mutations are also strongly associated with a poor prognosis in MDS.^7^ Conversely, the average VAF of *TET2*, *ZRSR2*, *EZH2* and *U2AF1* was similar between preprogression and postprogression samples (Supplementary Table 4). The VAF of a small number of mutations decreased in the postprogression samples, possibly owing to the dominant clone not being ancestral or to the emergence of competing subclones carrying mutations that were not detected by our gene panel.

The large majority of *TET2*, *ZRSR2* and *EZH2* mutations in preprogression samples had VAFs >40%, within a range that, according to previous studies that analyzed the clonal architecture of MDS and AML from WES or WGS data,^14, 15^ is consistent with these mutations being present in a founding clone. Six out of eight *TP53* mutations found in preprogression samples had VAFs smaller than 30% (range 8–27%), suggesting that mutations of this gene occurred mainly in a subclone; four of these subclonal mutations expanded with disease progression and two additional *TP53* mutations were present in postprogression samples only. Four *NRAS* mutations identified in preprogression samples were subclonal (VAF range 7–30%); eight *NRAS* mutations were present in postprogression samples only, suggesting that the emergence of new *NRAS* mutations during the course of the disease may have a role in disease progression. The VAF of all three subclonal mutations in *RUNX1* in preprogression samples (VAF range 12–19%) increased with disease progression; in addition, four *RUNX1* mutations were found in postprogression samples only, suggesting that in the case of *RUNX1* both the emergence of new mutations and the expansion of existing ones during the disease course may be involved in progression to AML.

An additional serial sample was sequenced for four cases in our cohort (Supplementary Figure 2). In one case, an *NRAS* mutation expanded during disease progression, whereas the allele burden of mutations in *ASXL1*, *RUNX1* and *EZH2* remained constant. In another case, within a background of mutations in *ASXL1*, *EZH2* and *ZRSR2*, a mutation in *SETBP1* emerged mid-progression and a mutation in *NRAS* was found at the AML stage only. This shows that more precise information on the mutational profile and subclone evolution during disease progression can be obtained by the analysis of multiple serial samples.

This is the first study to investigate the mutational status of a large group of MDS patients showing disease progression by the study of serial samples using a NGS myeloid gene panel. We have determined the frequency and chronology of myeloid gene mutation acquisition during disease progression in MDS, identifying specific mutations that are associated with disease evolution, and illuminating the role of subclone development in MDS progression. These data suggest that there are several genetic paths for MDS progression.

## Figures and Tables

**Figure 1:**
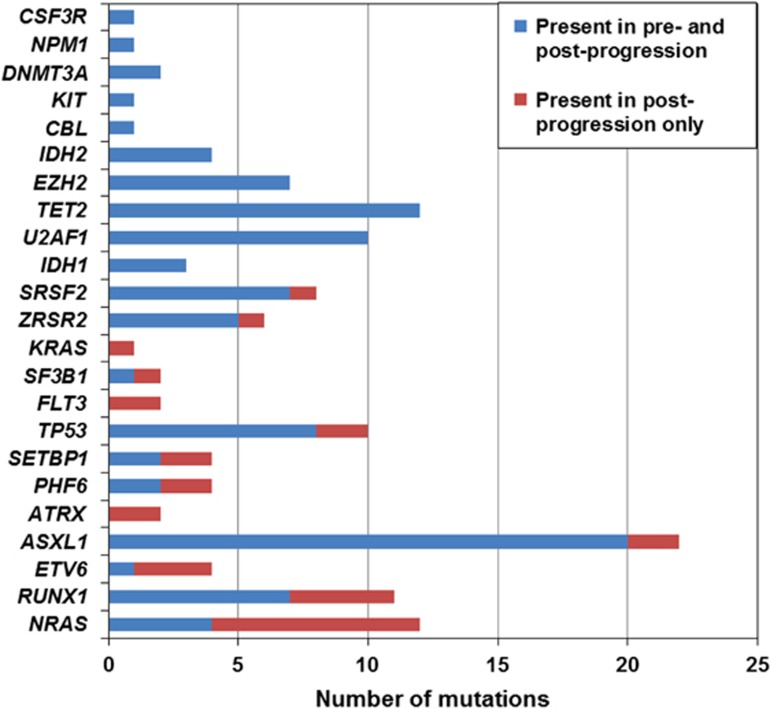
Timing of mutation occurrence in preprogression and postprogression samples.

**Table 1:** Number and percentage of mutated preprogression and postprogression samples for each gene

*Genes*	*Number (%) of mutated preprogression samples*	*Number (%) of mutated postprogression samples*
*ASXL1*	18 (44%)	19 (46%)
*TET2*	11 (27%)	11 (27%)
*U2AF1*	10 (24%)	8 (20%)
*RUNX1*	7 (17%)	11 (27%)
*TP53*	7 (17%)	7 (17%)
*SRSF2*	6 (15%)	7 (17%)
*EZH2*	5 (12%)	5 (12%)
*ZRSR2*	5 (12%)	6 (15%)
*IDH2*	4 (10%)	4 (10%)
*NRAS*	3 (7%)	8 (20%)
*IDH1*	3 (7%)	3 (7%)
*SETBP1*	2 (5%)	4 (10%)
*PHF6*	2 (5%)	3 (7%)
*DNMT3A*	2 (5%)	2 (5%)
*CBL*	1 (2%)	1 (2%)
*KIT*	1 (2%)	1 (2%)
*CSF3R*	1 (2%)	0 (0%)
*ETV6*	1 (2%)	4 (10%)
*SF3B1*	1 (2%)	2 (5%)
*NPM1*	1 (2%)	1 (2%)
*ATRX*	0 (0%)	2 (5%)
*KRAS*	0 (0%)	1 (2%)
*FLT3*	0 (0%)	2 (5%)
