# Supplementary material for: Targeted resequencing analysis of 31 genes commonly mutated in myeloid disorders in serial samples from myelodysplastic syndrome patients showing disease progression
Source: Leukemia. 2015 Jun 26;30(1):248–50. doi: 10.1038/leu.2015.129 (PMC4705423; doi:10.1038/leu.2015.129)
Supplement: Supplementary Information [file leu2015129x1.doc]

**Supplementary methods**

All samples were collected in accordance with local ethics committees after obtaining informed consent. The TruSeq Custom Amplicon (TSCA) panel was generated using the online DesignStudio pipeline (http://designstudio.illumina.com, Illumina), and covers a total of 36,242bp with 287 amplicons. In genes with well-defined mutational hotspots only these regions were targeted; otherwise the entire coding sequence of the gene was sequenced. Dual-barcoded TSCA libraries were created from 250ng of genomic DNA, in accordance with the manufacturer’s instructions, before undergoing 2x150bp paired-end sequencing on the Illumina MiSeq platform. Variants were annotated and filtered using Illumina VariantStudio v2.1.36 data analysis software (reference genome build hg19). Variants with a quality score >30, sequencing read depth >30 and variant allele frequency >5% were selected. All variants were visually inspected in Integrative Genomics Viewer (IGV, http://www.broadinstitute.org/igv/). Variants reported in dbSNP (www.ncbi.nlm.nih.gov/SNP), not reported in the Catalogue of Somatic Mutations in Cancer (COSMIC, http://cancer.sanger.ac.uk/cancergenome/projects/cosmic/) and scored as tolerated or benign by SIFT (http://sift.jcvi.org/) or Polyphen (http://genetics.bwh.harvard.edu/pph2/) were excluded. We then checked whether the filtered variants were previously reported in the literature.

In cases where a mutation was identified in only either the pre- or the post-progression sample for a particular patient, both the variant calling file and aligned sequencing reads of the paired sample were checked for presence of the mutation. If the mutation was present, albeit at a low VAF or sequencing depth, then it was reported in our data together with its sequencing coverage and VAF.

The coverage at the sites of the *SRSF2* mutations reported was found to be low and therefore the coverage and variant allele frequency for *SRSF2* mutations was determined by visual inspection using IGV. *SRSF2* mutations were confirmed using Sanger sequencing in nine samples with sufficient material remaining for the analysis. Variant allele frequency analysis was not performed for *SRSF2* mutations.
