# Supplementary material for: Targeted resequencing analysis of 31 genes commonly mutated in myeloid disorders in serial samples from myelodysplastic syndrome patients showing disease progression
Source: Leukemia. 2015 Jun 26;30(1):248–50. doi: 10.1038/leu.2015.129 (PMC4705423; doi:10.1038/leu.2015.129)
Supplement: Supplementary Table 2 [file leu2015129x3.doc]

**Supplementary Table 2.** List of the 31 genes targeted for enrichment in the TSCA library.

| *ASXL1* | *DNMT3A* | *JAK2* | *PDGFRA* | *SRSF2* |
| --- | --- | --- | --- | --- |
| *ATRX* | *ETV6* | *KIT* | *PHF6* | *TET2* |
| *CBL* | *EZH2* | *KRAS* | *PTEN* | *TP53* |
| *CBLB* | *FLT3* | *MPL* | *RUNX1* | *U2AF1* |
| *CBLC* | *HRAS* | *NPM1* | *SETBP1* | *WT1* |
| *CSF3R* | *IDH1 / IDH2* | *NRAS* | *SF3B1* | *ZRSR2* |
