# Supplementary material for: Targeted resequencing analysis of 31 genes commonly mutated in myeloid disorders in serial samples from myelodysplastic syndrome patients showing disease progression
Source: Leukemia. 2015 Jun 26;30(1):248–50. doi: 10.1038/leu.2015.129 (PMC4705423; doi:10.1038/leu.2015.129)
Supplement: Supplementary Table 3 [file leu2015129x4.doc]

**Supplementary Table 3.** Number of mutations per gene in pre-progression and post-progression samples.

| **Genes** | **No. of mutations in pre-progression samples** | **No. of mutation in post-progression samples** |
| --- | --- | --- |
| *ASXL1* | 20 | 22 |
| *TET2* | 12 | 12 |
| *U2AF1* | 10 | 8 |
| *RUNX1* | 7 | 11 |
| *TP53* | 8 | 8 |
| *SRSF2* | 7 | 7 |
| *EZH2* | 7 | 7 |
| *ZRSR2* | 5 | 6 |
| *NRAS* | 4 | 11 |
| *IDH2* | 4 | 4 |
| *SETBP1* | 2 | 4 |
| *IDH1* | 3 | 3 |
| *PHF6* | 2 | 3 |
| *ATRX* | 0 | 2 |
| *DNMT3A* | 2 | 2 |
| *KIT* | 1 | 1 |
| *CSF3R* | 1 | 0 |
| *ETV6* | 1 | 4 |
| *SF3B1* | 1 | 2 |
| *CBL* | 1 | 1 |
| *NPM1* | 1 | 1 |
| *KRAS* | 0 | 1 |
| *FLT3* | 0 | 2 |
| **Total** | **99** | **122** |
