# Supplementary material for: Targeted resequencing analysis of 31 genes commonly mutated in myeloid disorders in serial samples from myelodysplastic syndrome patients showing disease progression
Source: Leukemia. 2015 Jun 26;30(1):248–50. doi: 10.1038/leu.2015.129 (PMC4705423; doi:10.1038/leu.2015.129)
Supplement: Supplementary Table 4 [file leu2015129x5.doc]

**Supplementary Table 4.** Average VAF in pre- and post-progression samples for genes mutated in more than five cases.

| **Gene** | **Average VAF Pre-progression** | **Average VAF Post-progression** | **Ratio** |
| --- | --- | --- | --- |
| *TP53* | 26.63 | 40.92 | 1.54 |
| *NRAS* | 16.77 | 25.00 | 1.49 |
| *RUNX1* | 32.58 | 40.18 | 1.23 |
| *EZH2* | 53.38 | 60.54 | 1.13 |
| *ASXL1* | 31.38 | 35.03 | 1.12 |
| *TET2* | 48.73 | 51.45 | 1.06 |
| *ZRSR2* | 79.26 | 83.29 | 1.05 |
| *U2AF1* | 38.52 | 39.66 | 1.03 |
