# Supplementary material for: Targeted resequencing analysis of 31 genes commonly mutated in myeloid disorders in serial samples from myelodysplastic syndrome patients showing disease progression
Source: Leukemia. 2015 Jun 26;30(1):248–50. doi: 10.1038/leu.2015.129 (PMC4705423; doi:10.1038/leu.2015.129)

**Supplementary Figure 1.** Number of mutations per sample in pre-progression and post-progression samples.


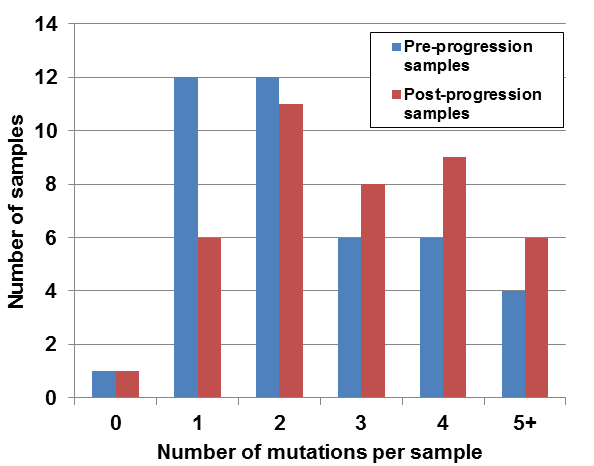

Supplement: Supplementary Figure 1 [file leu2015129x6.doc]
